# HIV and AIDS-related knowledge among women in Iraq

**DOI:** 10.1186/1756-0500-1-123

**Published:** 2008-12-01

**Authors:** Seter Siziya, Adamson S Muula, Emmanuel Rudatsikira

**Affiliations:** 1 Department of Community Medicine, University of Zambia Medical School, Lusaka, Zambia; 2 Department of Community Health, University of Malawi College of Medicine, Blantyre, Malawi; 3 Division of Epidemiology and Biostatistics, Graduate School of Public Health, San Diego State University, San Diego, California, USA

## Abstract

**Background:**

Individuals who are aware of the risk of infection and perceive themselves to be at risk of infection are more likely to take action to prevent HIV infection. The aim of this study was to assess the knowledge of HIV/AIDS among Iraqi women.

**Methods:**

A secondary analysis of the 2000 Multiple Cluster Indicator Survey (MICS) for Iraq was carried out to assess the extent of HIV/AIDS-related knowledge among Iraqi women.

**Results:**

The majority of the 22,997 respondents were age 15–24 years (44.3%), currently married (51.4%), and resided in urban areas (71.7%). About 1 in 4 (26.0%) of the study participants had no formal education. Only 49.9% had heard of HIV/AIDS. Overall, 60.5% did not know that HIV can be transmitted through blood transfusion. Meanwhile, 98.5% of the respondents did not know that HIV can be transmitted from mother to child through breast milk. Only 0.7% of the respondents reported that HIV cannot be transmitted through mosquito bites. The proportion of the respondents who had adequate knowledge on HIV/AIDS was 9.8%. Adequate knowledge of HIV/AIDS was negatively associated with being married, poor, having low education, and residing in rural areas.

**Conclusion:**

Findings from this study indicate that adequate knowledge of HIV/AIDS among Iraqi is very limited and associated with marital status, education, wealth, and place of residence. This information may be of use in the design, targeting, monitoring and evaluation of programs aimed at improving HIV and AIDS related knowledge in Iraq.

## Background

Reports suggest that the human immune-deficiency virus pandemic largely spared Middle Eastern countries in the first one and a half decades. To some extent, this perception was facilitated by lack of reliable data for much of the region during the period. Data on HIV prevalence, the determinants of transmission, treatment, care and support for infected persons in Iraq are limited. This is probably understandable considering the difficult socio-political environment that the country has gone through since the end of the Gulf War in 1991.

The Iraqi "Epidemiological Fact Sheet on HIV and AIDS, Core data on epidemiology and response" from the UNAIDS exemplifies the paucity of data in that it has limited information compared to other countries in the Middle East region [1]. While HIV prevalence in the region is generally lower than has been reported in southern Africa [2,3], basing policy and programmatic decisions on account of information from regional data or data from neighboring countries may not be the most appropriate approach. There is therefore urgent need for country level data for most of the countries in the Middle Eastern Region.

Individuals who are aware of the risk of infection and perceive themselves to be at risk of infection are more likely to take action to prevent HIV infection [4-6]. If this assumption were true in the case of HIV infection in Iraq, we would expect that lack of adequate knowledge on the modes of HIV transmission would lead to failure to take preventive measures and consequently result in acquisition of infection.

Using the 2000 Multiple Cluster Indicator Survey (MICS) for Iraq, we estimated the prevalence of adequate knowledge on HIV among a sample of adult women. We also assessed whether knowledge on HIV was associated with a number of socio-demographic variables: wealth; age; education; marital status and; residence status (rural versus urban). We believe this information may be of use in the design, targeting, monitoring and evaluation of programs aimed at improving HIV and AIDS related knowledge in Iraq.

## Methods

### Sample Design

A three stage sampling procedure was used in the identification of eligible study participants. In the first stage of sampling villages or *Mahallas *in each sub-district (Qada'a) were listed together with their population estimates. Mahallas were selected with the probability of being selected proportional to their population. In the second stage, each mahalla was divided into compartments with population of about 500 people. One or more compartments were then selected from a village. Then each compartment was divided into blocks or *Majals *with 25–30 households in urban area and 20–25 households in rural areas. One *Majal *was then selected by simple random sampling. In the final stage of sampling, each selected *Majal *an update of existing household listing was carried out and a cluster of 10 households was selected by systematic random sampling. Recruitment occurred in all the 18 governorates of country; with each governorate recruiting 740 households except Baghdad with a sample size of 850 households.

### Questionnaire

The MICS questionnaire modeled the generic from UNICEF was used. An Arabic translation core questionnaire, provided by the UNICEF's Middle East and North Africa Regional Office (MENARO) was revised and adapted to be culturally and linguistically acceptable. Pre-testing of the questionnaire and modifications were made as necessary. A description of the MICS methodology has been reported elsewhere [7-9].

### Data analysis

Data were analysed using the Statistical Package for the Social Sciences (SPSS) version 11.5 (Chicago, Illinois, United States). We estimated the prevalence of reporting whether study participants had ever heard about HIV/AIDS, and whether they reported a "correct" answer to the following: whether AIDS can be avoided; whether they believed that transmission is possible via sexual intercourse, contaminated equipment, blood transfusion, breast milk, mosquito bites and whether a pregnant woman who is infected can transmit infection to her unborn child. We also performed both bivariate and multivariate logistic regression to assess whether a list of explanatory variables (wealth; age; education; marital status and; rural versus urban residence were associated with having adequate HIV-related knowledge. For the purpose of this study, adequate knowledge was defined as having reported correct answers on 6 out of 8 questions listed in Table 1. We present unadjusted OR (95%CI) from the bivariate analysis and AOR (95%CI) from the multivariable analysis. Although no response on any of the questions may have meant that the respondents did not know the correct answer, it is also plausible to suggest that may be they were uncomfortable to deal with the issue. Therefore, the analysis was based on those who responded as "yes" or "no", and the rest of responses were recoded as missing information.

**Table 1:** Distributions of levels of HIV/AIDS-related knowledge among women in Iraq

Factor	N* (%)**
Heard of HIV/AIDS	
no	12853 (50.1)
yes	10131 (49.9)
	
AIDS can be avoided	
no	1396 (14.8)
yes	7266 (85.2)
	
HIV can be transmitted through sexual intercourse	
no	893 (11.5)
yes	6372 (88.5)
	
HIV can be transmitted through blood transfusion	
no	4249 (60.5)
yes	2442 (39.5)
	
HIV can be transmitted from mother to child during pregnancy	
no	6276 (95.7)
yes	309 (4.3)
	
HIV can be transmitted from mother to child through breast milk	
no	6458 (98.5)
yes	104 (1.5)
	
HIV can be transmitted through mosquito bites	
no	61 (0.7)
yes	6493 (99.3)
	
HIV can be transmitted through contaminated equipment	
no	5637 (87.1)
yes	944 (12.9)
	
Total knowledge (number of correct items out of a possible 8)	
2	14 (0.2)
3	648 (8.6)
4	3429 (51.2)
5	1738 (30.2)
6	596 (8.3)
7	74 (1.0)
8	38 (0.5)

* unweighted frequency ** weighted percent

### Human subjects' research

The Iraqi MICS survey protocol was approved by the country's Central Statistical Organization. Our analysis involves secondary analysis of de-indentified data obtained from the UNICEF.

## Results

Altogether there were 22,997 women who participated in the survey. The majority of the respondents were age 15–24 years (44.3%), currently married (51.4%), and resided in urban areas (71.7%). About 1 in 4 (26.0%) of the respondents had not been to formal school. Further description of the sample is shown in Table 2.

**Table 2:** Socio-demographic characteristics of the respondents in the Iraq MICS 2000

Factor	n* (%**)
Age (years)	
15–24	10327 (44.3)
25–34	6730 (29.3)
35–49	5940 (26.4)
	
Marital status	
currently married	11924 (51.4)
formerly married	1107 (4.9)
never married	9966 (43.7)
	
Maternal education level attained	
none	7032 (26.0)
primary	9094 (38.0)
secondary or higher	6533 (34.7)
non-standard curriculum	332 (1.3)
	
Wealth index (quintiles)	
poorest	6147 (19.0)
second	4810 (20.3)
middle	4682 (19.5)
fourth	3686 (24.3)
richest	3648 (16.9)
	
Area	
urban	13946 (71.7)
rural	9027 (28.3)

* unweighted frequency ** weighted percent

Table 1 shows levels of knowledge on HIV/AIDS-related items. Only 49.9% had heard of HIV/AIDS. Overall, 60.5% did not know that HIV can be transmitted through blood transfusion. Meanwhile, 98.5% of the respondents did not know that HIV can be transmitted from mother to child through breast milk. Only 0.7% of the respondents reported that HIV cannot be transmitted through mosquito bites. The proportion of the respondents who had adequate knowledge on HIV/AIDS was 9.8%.

While age was significantly associated with HIV/AIDS-related knowledge in bivariate analysis, it was not the case in multivariate analysis (Table 3). Marital status, education, wealth and area of residence were all significantly associated with knowledge at both bivariate and multivariate analyses. Compared to respondents who were never married, those who were currently married were 18% (AOR = 0.82, 95%CI [0.72, 0.94]) less likely to have adequate knowledge, while those who were formerly married were 50% (AOR = 1.50, 95%CI [1.20, 1.86]) more likely to have adequate knowledge. Respondents without formal education were 49% (AOR = 0.51, 95%CI [0.31, 0.86]) less likely to have adequate knowledge compared to those who had non-standard curriculum schooling. Meanwhile, respondents who had secondary or higher level of education were 2.23 (95%CI [1.56, 3.19]) times more likely to have adequate knowledge compared to those who had non-standard curriculum schooling. Compared to the respondents in the richest quintile, those in the second and fourth quintile were less likely to have adequate knowledge (AOR = 0.78, 95%CI [0.62, 0.99] and (AOR = 0.71, 95%CI [0.59, 0.86], respectively). Meanwhile, respondents in the middle quintile were 65% (AOR = 1.65, 95%CI [1.37, 1.98]) more likely to have adequate knowledge compared to those in the richest quintile. Finally, respondents in the urban areas were 24% (AOR = 1.24, 95%CI [1.02, 1.51]) more likely to have adequate knowledge compared to those who lived in rural areas.

**Table 3:** Correlates of HIV/AIDS-related knowledge among women in Iraq

Factor	OR* (95%CI)	AOR** (95%CI)
Age (years)		
15–24	1.05 (0.85, 1.16)	-
25–34	0.94 (0.84, 1.04)	
35–49	1	
		
Marital status		
currently married	0.78 (0.68, 0.89)	0.82 (0.72, 0.94)
formerly married	1.41 (1.15, 1.75)	1.50 (1.20, 1.86)
never married	1	1
		
Maternal education level attained		
none	0.55 (0.33, 0.92)	0.51 (0.31, 0.86)
primary	0.95 (0.66, 1.39)	0.91 (0.63, 1.33)
secondary or higher	2.45 (1.73, 3.48)	2.23 (1.56, 3.190
non-standard curriculum	1	1
		
Wealth index (quintiles)		
poorest	0.45 (0.29, 0.70)	0.73 (0.45, 1.20)
second	0.75 (0.59, 0.95)	0.78 (0.62, 0.99)
middle	1.74 (1.46, 2.08)	1.65 (1.37, 1.98)
fourth	0.89 (0.74, 1.06)	0.71 (0.59, 0.86)
richest	1	1
		
Area		
urban	1.56 (1.31, 1.86)	1.24 (1.02, 1.51)
rural	1	1

OR* unadjusted odds ratio AOR** adjusted odds ratio

## Discussion

In a study of Iraqi women in 2000, just about half (49.9%) reported to have heard about HIV/AIDS. Interestingly however, more than half believed that HIV could be avoided and could be transmitted via sexual intercourse i.e. 85.2% and 88.2% respectively. This discrepancy in that a smaller proportion reported having ever heard about HIV/AIDS and yet a larger proportion reported knowing how HIV could be transmitted and that it was possible to be avoided, deserves further inquiry. It is possible that a much higher proportion had ever heard about HIV than reported having heard so, or a much larger proportion guess the responses to transmission and prevention. The proportions of women who report having heard about HIV/AIDS, though high is much lower than that reported in the Malawi, Zambia and Zimbabwe Demographic Health Surveys (conducted in 2004, 2001/2 and 2005/6 respectively) where knowledge was almost universal [10-12]. An obvious difference between these African countries and the Middle East is that southern African is the "epicentre" of the world AIDS epidemic and so, interest in HIV by health programs and the general community is especially high.

It is noteworthy that 39.5% and 12.9% reported knowing that HIV could be transmitted by blood transfusion and contaminated equipment. While it can be argued that the general population deserves to know about the possible adverse effects following blood transfusion, in the main however, many patients may not be in a position to prevent transfusion when they need it for a diversity of medical indications. The UNAIDS reports that unprotected paid sex and the use of contaminated drug injecting equipment are the main modes of HIV spread in North Africa and the Middle East [1]. Failure to appreciate the importance of contaminated injecting equipment may expose women who are injecting drug users to HIV infections. Furthermore, women who have partners who are injecting drug users may also fail to appreciate the potential risk to HIV infection that their partners expose them to. Understanding that blood transfusion may expose individuals to HIV infection may be more useful at the policy stage where a health system needs to implement universal blood HIV screening mandatory.

Only a small proportions of women believed that an HIV infected woman could transmit HIV to her unborn child and during breast feeding (4.3% and 1.5% respectively). In a study reported by Sandgren et al [13] on Kazakhstan, only 46% and 68% of the women reported that breastfeeding and mother-to-child transmission during pregnancy or delivery were believed to be routes of HIV transmission. In an environment where HIV infection among women is very low, such lack of knowledge may not be particularly concerning. However, for the small number of women who are HIV infected, accurate information would potentially influence their families' reproductive decisions and breastfeeding options in the event that they have baby.

Almost all women (99.3%) believed that mosquitoes could transmit HIV. Mazloomy and Baghianimoghadam [14] reported 20.3% teachers in Iraq thought that mosquitoes could transmit HIV/AIDS. In Papua New Guinea, Andersson et al [15] reported that 36% of women attending prenatal care in Port Moresby believed that HIV can be spread by mosquitoes. While the correct information is that mosquitoes do not transmit HIV, we are not sure what the implications of the myth are. One the one hand, if people avoid mosquito bites even in the belief that HIV could be avoided in that way, public health gains may be achieved. On the other hand, if people believed that nothing can be done to prevent HIV infections because no matter what one does, at some point they are going to be bitten by a mosquito, then there is a problem. However, even though there may not be significant public health benefits from the correct knowledge, we believe that the belief that mosquitoes spread HIV is just an important indication that myths about HIV transmissions are high in Iraq.

In multivariable logistic regression, we found that urban residence, having been formerly married, having had secondary education or more, were associated with having correct knowledge on HIV. Except for the middle wealth quintile, all wealth categories had were less likely to have correct HIV information compared to the highest wealth quintile, thus suggesting that lower socioeconomic status (measured by wealth status) was associated with inadequate knowledge. Although we do not have information as to why formerly married women could have higher HIV knowledge than other women, the results deserve. It is possible that these women may have been no longer married as a result of being widowed or having been divorced. In sub-Sahara Africa, widowed and divorced women are more likely to be HIV infected than other women. If marital dissolution were more likely to be associated with infidelity, it may be understandable that women in such information may be more likely to seek information on HIV and AIDS. Ramezani Tehrani and Malek-Afzali [16] in a study from Iran, reported that individuals at higher risk of HIV (sex workers, truck drivers) were more likely to be more knowledgeable on HIV and HIV. Their higher knowledge compared to low-risk sub-population had little to do with public awareness efforts, rather individual efforts. The implications of the above findings are that HIV information programs should not neglect rural areas and people in low socio-economic status.

Despite the strength that the results are likely to be representative of the national situation, this study has a number of limitations. Firstly, data were cross sectional and therefore the measures of effect (odds ratios) signify associations and not causation [17]. Secondly, to the extent that the study participants misreported, either intentional or inadvertently, our findings may be biased. The fact that no personal identifiers were reported may have potentially removed the need for purposeful misreporting. Perhaps more importantly, the prevalence of the various characteristics represents the situation at the time in 2000. The Iraq socio-political environment has changed significantly from that time to the present. Finally, while many of the associations found between the outcome (HIV knowledge) may have changed over the years; this would make our findings of limited contemporary application.

## Conclusion

Findings from this study indicate that adequate knowledge of HIV/AIDS among Iraqi is very limited and associated with marital status, education, wealth, and place of residence. This information may be of use in the design, targeting, monitoring and evaluation of programs aimed at improving HIV and AIDS related knowledge in Iraq.

## Competing interests

The authors declare that they have no competing interests.

## Authors' contributions

SS conducted data analysis, participated in the interpretation of the results and drafting of the manuscript. ASM participated in the interpretation of the results and drafting of the manuscript. ER participated in the interpretation of the results and drafting of the manuscript.
